# Efficacy and safety of trastuzumab emtansine (T-DM1) in the treatment of HER2-positive metastatic breast cancer (MBC): a meta-analysis of randomized controlled trial

**DOI:** 10.18632/oncotarget.22270

**Published:** 2017-11-01

**Authors:** Hongjing Yan, Kewei Yu, Kaile Zhang, Linxia Liu, Yue Li

**Affiliations:** ^1^ Department of Microbial Testing, Minhang District Centers for Disease Control and Prevention, Shanghai 201101, China; ^2^ Department of Rehabilitation Medicine, Huashan Hospital, Fudan University, Shanghai 200040, China; ^3^ Department of Urology, Shanghai Sixth People's Hospital, Shanghai 200233, China; ^4^ Shanghai University of Medicine and Health Sciences, Shanghai 201318, China; ^5^ Department of Immunization Program, Hongkou District Centers for Disease Control and Prevention, Shanghai 200082, China

**Keywords:** trastuzumab emtansine, HER2-positive, breast cancer, meta-analysis

## Abstract

**Aims:**

Trastuzumab emtansine (T-DM1), an antibody-drug conjugate against human epidermal growth factor receptor 2 (HER2), has been used in the treatment of patients with HER2-positive metastatic breast cancer (MBC). We conducted a meta-analysis to evaluate the efficacy and toxicity of T-DM1 for the treatment of patients with HER2-positive MBC.

**Materials and Methods:**

Randomized controlled trials (RCTs), published in Pubmed, Embase, and Web of Science were systematically reviewed to assess the survival benefits and toxicity profile of HER2-positive patients with MBC who were treated with T-DM1. Outcomes included progression-free survival (PFS), overall survival (OS), overall response rate (ORR), and toxicities. Results were expressed as the hazard ratio (HR) with 95% confidence intervals (CIs).

**Results:**

A total of 5 RCTs involving 3,720 patients met the inclusion criteria and were included in this meta-analysis. T-DM1 significantly prolonged PFS (HR = 0.73, 95% CI: 0.61, 0.86; *P* < 0.05), OS (HR = 0.68, 95% CI: 0.62, 0.74; *P* < 0.05), but it did not increase ORR (RR = 1.25, 95% CI: 0.94, 1.66; *P* = 0.148). Subgroup analysis indicated that T-DM1 significantly improved PFS when it was used as first-line (HR = 0.86, 95% CI: 0.74, 1.00; *P* < 0.05) or non-first-line treatment (HR = 0.65, 95% CI: 0.53, 0.81; *P* < 0.05). T-DM1 was associated with more frequent adverse events, including fatigue, elevated ALT, elevated AST, and thrombocytopenia, than other anti-HER2 therapies.

**Conclusions:**

Based on the current evidence, T-DM1 significantly prolonged PFS and OS with a tolerated toxicity than other anti-HER2 therapies in patients with HER2-positive MBC. These findings confirm the use of T-DM1 for the treatment of patients with HER2-positive MBC. Further well-designed, multi-center RCTs needed to identify these findings.

## INTRODUCTION

Application of the human epidermal growth factor receptor 2 (HER2) gene occurs in approximately 20% to 25% of primary breast cancers and is associated with poor clinical outcomes in the absence of systemic therapy [1, 2]. The humanized HER2-targeted antibody trastuzumab (Herceptin; Genentech, South San Francisco CA), could improve survival of patients with HER2-positive metastatic breast cancer (MBC), when it is combined with standard chemotherapy [3, 4]. In spite of the efficacy of trastuzumab, most patients with HER2-positive develop progressive disease during or after trastuzumab treatment. Evidence from clinical practice shows that, HER2 over-expression persists and remains beyond progression [5–7], therefore, new strategies that changing the HER2-directed agent or switching chemotherapies in subsequent lines of treatment have been developed [8]. And now, there is no standard HER2-directed regimen approved for these heavily pretreated patients [9], and additional HER2-directed therapies are needed.

Trastuzumab emtansine (T-DM1) is an antibody-drug conjugate that incorporates the HER2-targeting properties of trasuzumab with the cytotoxic activity of the microtubule-inhibitory agent DM1 (derivative of maytansine) [10–12]. Trastuzumab and DM1 are covalently conjugated by means of a stable linkers [13, 14]. T-DM1 could improve the therapeutic index and limit the exposure of normal tissue through the delivering the intracellular drug specifically to HER2-overexpressing cells. T-DM1, as a single-agent treatment for patients with HER2-positive MBC who were previously treated with trastuzumab and a concurrent or sequential taxane, was recently approved in the USA and European Union. Results from several phase 2 studies show that T-DM1 was clinical effective in the treatment of patients with HER2-positive advanced or metastatic breast cancer [15–17]. These impressive results have provided a strong rationale for conducting randomized controlled trails (RCTs) that assess T-DM1 for HER2-positive breast cancer. In this study, we conducted a meta-analysis of these RCTs to evaluate the efficacy and safety of T-DM1, as compared with other anti-HER2 therapies, for HER2-positive breast cancer patients.

## RESULTS

### Identification of eligible studies

**Figure 1:**
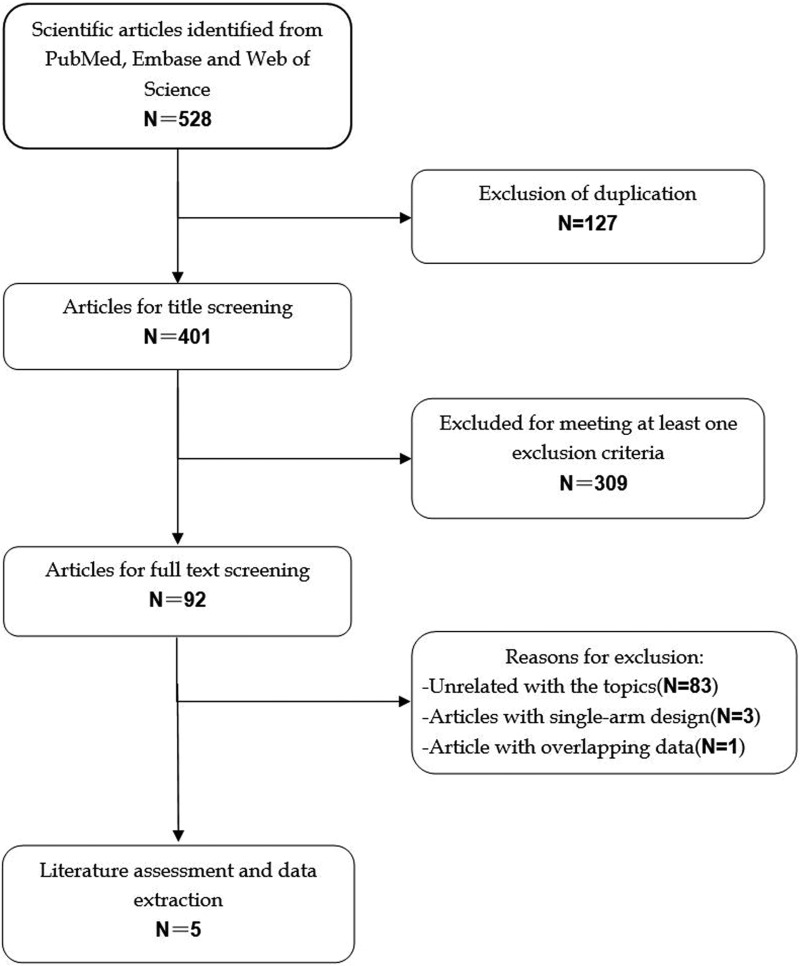
Search strategy and flow chart for this meta-analysis

The initial search yielded 528 relevant citations from Pubmed, Web of Science, and Embase. Of these, 127 were excluded as duplicate records, and 309 and 83 were excluded after review of title/abstract and full-text information, respectively (Figure 1). Therefore, 9 potential studies were identified for the final analysis; however, three studies were excluded because they were single-arm phase II studies [15–17], and one was excluded because it presented overlapping data with another study [18]. Finally, five RCTs (involving 3,720 patients) [19–23] that met the inclusion criteria were included in this meta-analysis. The Cohen statistic K for agreement on study inclusion was 0.92.

### Characteristics of eligible studies

The main patient characteristics of the four included studies were presented in Table 1. All five included studies were well-performed, prospective randomized controlled trials. Clinical characteristics were matched for age, estrogen receptor (ER)/ progesterone receptor (PR) status, and Eastern Cooperative Oncology Group performance status (ECOG PS) in each study. These studies were published between 2012 and 2017. Most of patients in these studies were from Europe, the Americas, and Asia. All these eligible patients were older than 18 years and had histologically or cytologically confirmed, HER2-positive, unresectable locally advanced breast or MBC. Among the five included studies, patients in three studies were previously treated with trastuzumab-based chemotherapy [19, 21], whereas patients in the remaining studies were treated with T-DM1 or trastuzumab plus docetaxel as first-line treatment [20, 22, 23]. In the T-DM1 group, patients were given a dose of 3.6 mg/kg intravenously once every 3 weeks.

**Table 1:** Baseline characteristics of patients in the trials included in the meta-analysis

Study	Treatment regimens	No. of patients	Median age (range, years)	ECOG PS	Hormone-receptor status	Median follow-up (range, months)
Verma S [19]	T-DM1	495	53 (25–84)	0/1:299/194	ER (+), PR (+), or both/ ER (–) and PR (–): 282/202	19.1 (0–40)
	Lapatinib + capecitabine	496	53 (24–83)	0/1:312/176	ER (+), PR (+), or both/ ER (–) and PR (–): 263/224	18.6 (0–41)
Hurvitz SA [20]	T-DM1	67	55 (27-–82)	0/1:44/23	ER (+), PR (+), or both/ ER (–) and PR (–): 33/34	23
	Trastuzumab + docetaxel	70	52 (33–75)	0/1:47/23	ER (+), PR (+), or both/ ER (–) and PR (–): 38/32	23
Welslau M [21]	T-DM1	450	NR	NR	NR	NR
	Lapatinib+ capecitabine	445	NR	NR	NR	NR
Krop IE [22]	T-DM1	404	NR	0/1/2:180/200/22	ER (+), PR (+), or both/ ER (–) and PR (–): 208/185	7.2 (5.0–10.1)
	Physician's choice	198	NR	0/1/2:82/101/15	ER (+), PR (+), or both/ ER (–) and PR (–): 103/85	6.5 (4.1–9.7)
Perez E. A [23]	T-DM1	367	52 (27–82)	0/1:239/128	ER (+) and/or PR (+)/ ER (–) and PR (–):195/160	35
	Trastumab+taxane	365	55 (22–88)	0/1:245/119	ER (+) and/or PR (+)/ ER (–) and PR (–):207/154	35
	T-DM1+pertuzumab	363	52 (27–86)	0/1:235/127	ER (+) and/or PR (+)/ ER (–) and PR (–):198/156	

Abbreviations: ES, estrogen receptor; PR, progesterone receptor; T-DM1, trastuzumab emtansine; ECOG PS, Eastern Cooperative Oncology Group performance status; NR, not reported.

Notably, in the TH3RESA [22] trial, patients were randomly assigned to T-DM1 or physician's choice. Of the physician's choice, about 83% of them were combination therapy with HER2-directed agent, including trastuzumab plus chemotherapy, and trastuzumab plus lapatinib.

### Quality assessment

**Figure 2:**
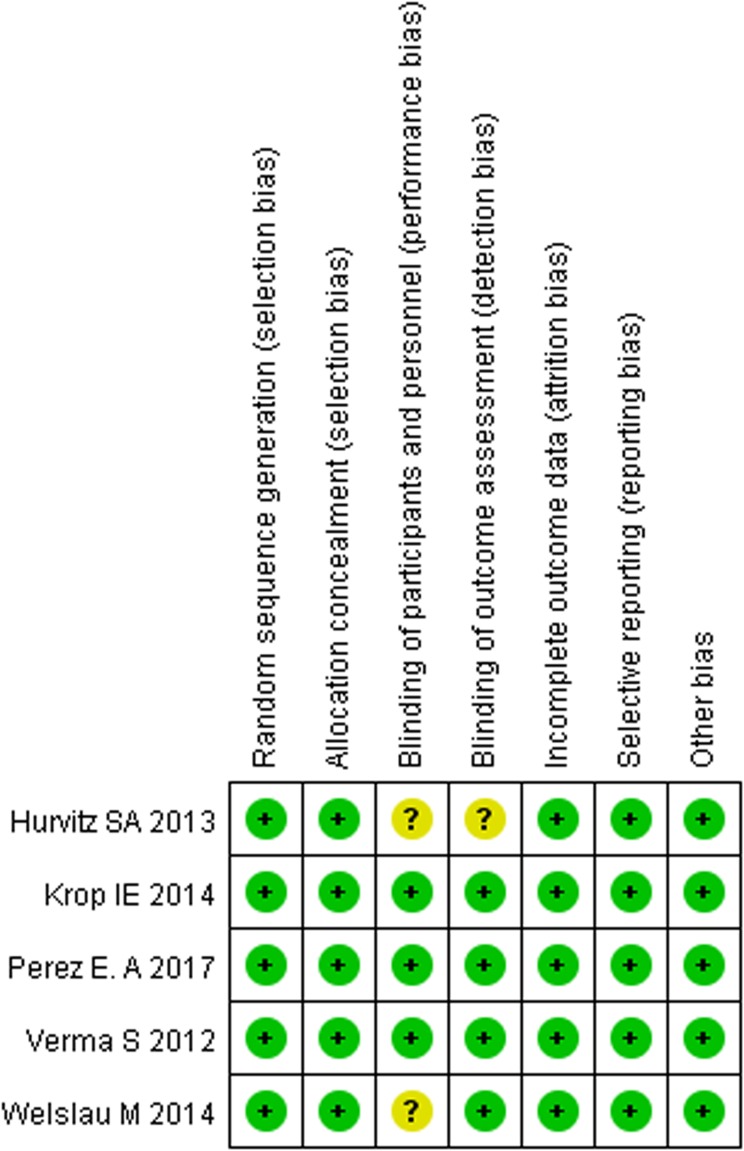
Risk of bias summary

The details of risk bias are summarized in Figure 2. Overall, three trials were classified as being at low risk of bias [19, 22, 23], and two as being at unclear risk of bias [20, 21]. The main reason for the two trials with unclear risk of bias was that the blinding of outcome assessments was unclear or seldom reported. The adequate randomized sequence, and appropriate allocation concealment were reported in all the included trials [19–23]. There were incomplete outcome data or selective reporting, or other bias in all the included trials [19–23].

### Progression-free survival

**Figure 3:**
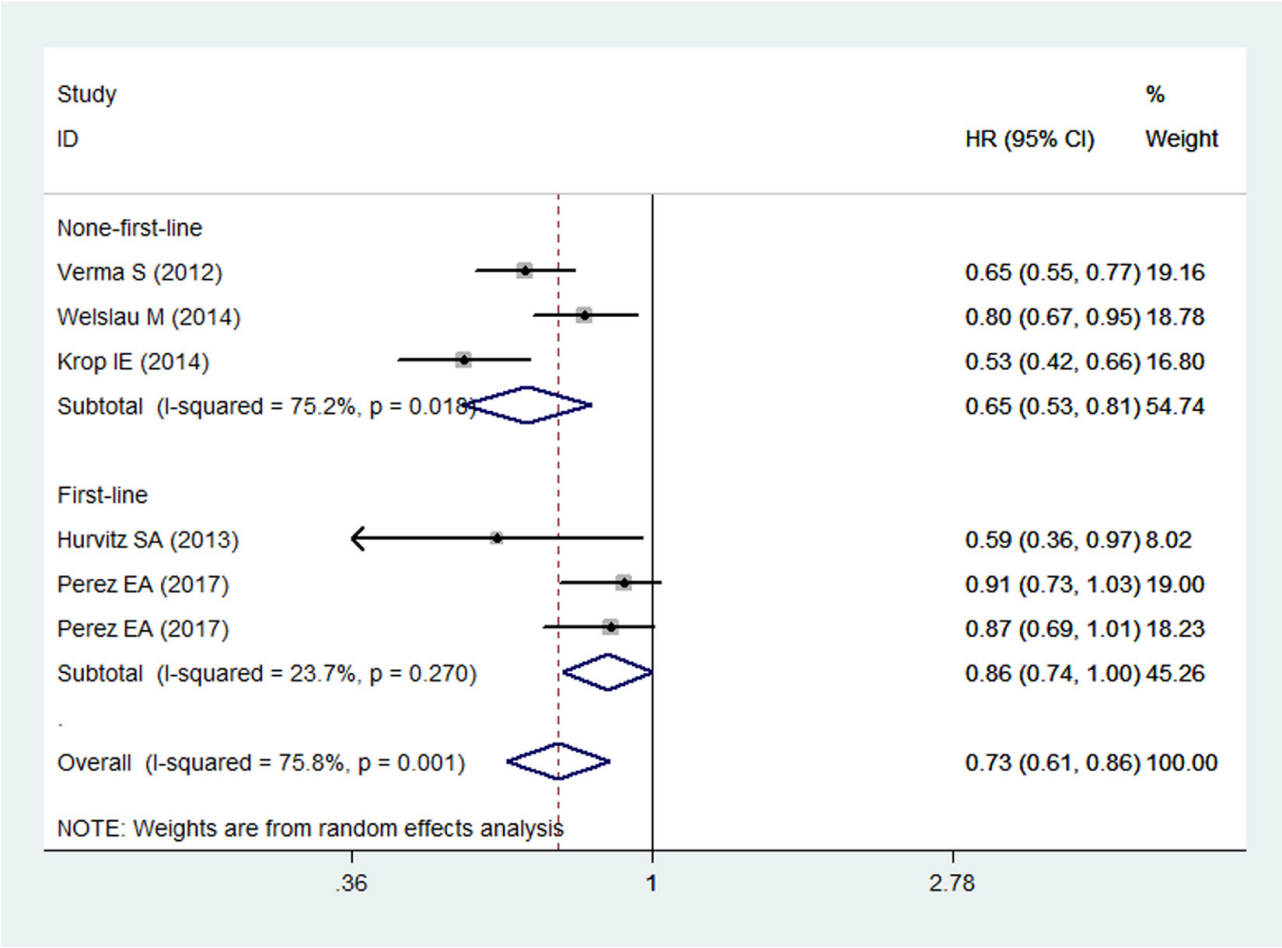
Comparison of T-DM1 with other chemotherapies for HER2-positive patients with MBC in terms of progression free survival (PFS)

All five RCTs reported PFS in study patients [19–23]. The pooled results of these studies show that T-DM1 significantly prolonged the PFS in patients with HER2-positive MBC (HR = 0.73, 95% CI: 0.61, 0.86; *P* < 0.05) (Figure 3). The test for heterogeneity was significant (P for heterogeneity = 0.001; I^2^ = 75.8%). Therefore, we performed sensitivity analysis to explore potential sources of heterogeneity. When we excluded the trial conducted by Krop IE et al. [22], the heterogeneity was resolved (I^2^ = 45.6%, *P* = 0.347), and the result changed slightly (HR = 0.78, 95% CI: 0.68, 0.90, *P* < 0.05), which indicated that this study probably contributed to the heterogeneity.

We also performed subgroup analysis to evaluate the impact of different treatment line on the overall estimation. The results revealed that T-DM1 was associated with an increased PFS in the patients with HER2-positive MBC no matter it was used as first-line (HR = 0.86, 95% CI: 0.74, 1.00; *P* < 0.05) or non-first-line treatment (HR = 0.65, 95% CI: 0.53, 0.81; *P* < 0.05) (Figure 3).

### Overall survival

**Figure 4:**
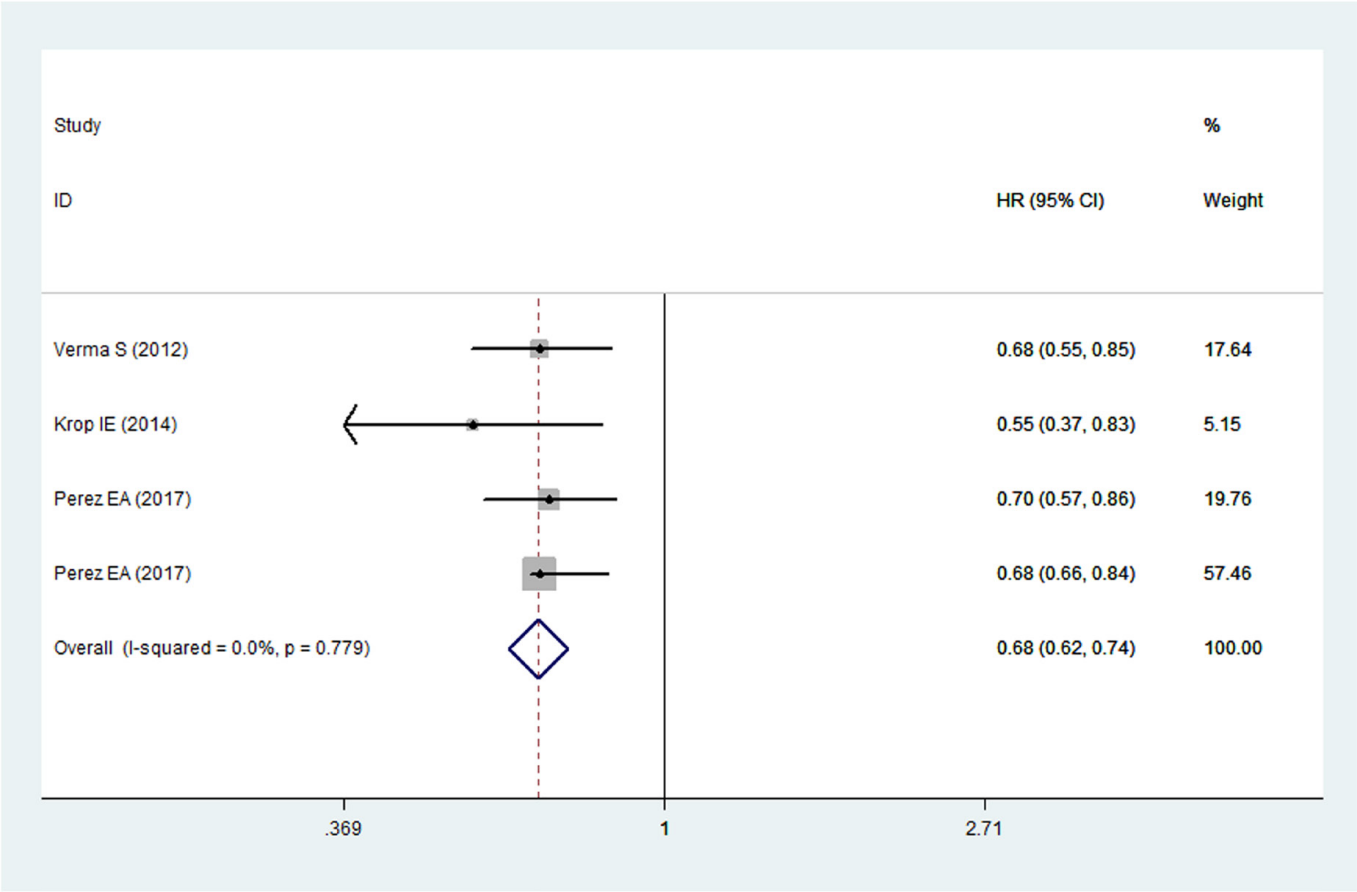
Comparison of T-DM1 with other chemotherapies for HER2-positive patients with MBC in terms of overall survival (OS)

Four RCTs reported the data of OS in patients [19, 22, 23]. The aggregated results suggest a significant improvement in OS between patients who received T-DM1 and those who received other anti-HER2 therapies (HR = 0.68, 95% CI: 0.62, 0.74; *P* < 0.05) (Figure 4). No statistical heterogeneity was observed between individual trials (P for heterogeneity = 0.779; I^2^ = 0.0%) (Figure 4).

### Overall response rate

**Figure 5:**
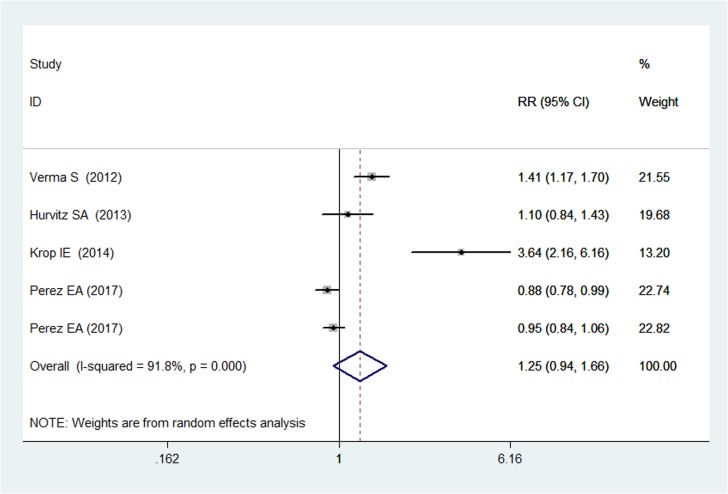
Comparison of T-DM1 with other chemotherapies for HER2-positive patients with MBC in terms of overall response rate (ORR)

Four RCTs reported the data on ORR [19, 20, 22, 23]. The pooled estimates showed that T-DM1 was associated with a similar ORR with other anti-HER2 therapies (RR = 1.25, 95% CI: 0.94, 1.66; *P* = 0.148) (Figure 5). There was statistical heterogeneity between individual trials (P for heterogeneity < 0.05; I^2^ = 91.8%) (Figure 5).

### Adverse events

All studies included in this meta-analysis presented data on adverse events [19–23]. Pooled analysis showed that compared to other anti-HER2 therapies, T-DM1 was associated with a significantly higher rate of fatigue, elevated ALT, elevated AST, and thrombocytopenia, but a significantly lower rate of diarrhea, vomiting, neutropenia, leucopenia, and febrile neutropenia (Table 2).

**Table 2:** Summary of the risk ration (RR) of adverse events in HER2-positive patients with MBC

Adverse events	RR	95% CI	*P* value
Fatigue	1.19	1.03, 1.37	0.021
Elevated ALT	2.47	1.19, 5.16	0.016
Elevated AST	2.68	1.40, 5.14	0.003
Thrombocytopenia	7.46	4.06, 13.70	0.000
Diarrhea	0.34	0.26, 0.47	0.000
Vomiting	0.72	0.51, 1.00	0.000
Neutropenia	0.35	0.18, 0.71	0.049
Leukopenia	0.25	0.08, 0.75	0.003
Febrile neutropenia	0.06	0.01, 0.32	0.014
Nausea	0.94	0.76, 1.16	0.541
Anemia	0.87	0.51, 1.49	0.612
Dyspnoea	0.78	0.39, 1.56	0.485

Abbreviations: ALT, alanine aminotransferase; AST, aspartate aminotransferase.

### Publication bias

Assessment of publication bias using Egger's and Begg's test showed that there was no potential publication bias among the included studies (Egger's test, *P* = 0.374; Begg's test, *P* = 0.463).

## DISCUSSION

The objective of this meta-analysis to evaluate the efficacy and safety of T-DM1 for HER2-positive patients with MBC. Our meta-analysis suggests that T-DM1 significantly prolonged PFS and OS, but did not increase the ORR. In addition, patients who received T-DM1 treatment exhibited a higher incidence of adverse events, including fatigue, elevated ALT, elevated AST, and thrombocytopenia, compared with those received other anti-HER2 therapies. These results confirmed the significant survival benefits of T-DM1 for HER2-positive MBC.

There have been two published systematic review and meta-analysis of T-DM1 for HER2-positive patients with MBC [24, 25]. Our study expends on the prior studies in providing more significant evidence for the use of T-DM1 in HER2-positive MBC. First, the present meta-analysis had a more enlarged sample sizes than the previous analysis, which enhanced the statistical power to assess this effect. In this meta-analysis, we included five RCTs, and all of them were prospective, randomized controlled phase 2/3 clinical trials. Whereas, in the previous studies, three were only two or three RCTs [25], and the number of contributing data was only two or three. Second, in this study, we used a fixed-effects or random-effects model to pool the data of included studies. With the method of meta-analysis, we were able to systematically summarize the current original studies on the effects of T-DM1, and provide some implications for future researches and decision making. Whereas, in the previous two studies, one was a systematic review [24], and no pooled data were provided. Thus, whether T-DM1 had advantaged survival effects than other treatments still remained uncertain. Third, in this meta-analysis, we also conducted subgroup analysis to evaluate the impact of different treatment line on the overall estimations, which was not analyzed in the prior meta-analysis [24, 25]. Fourth, in this study, we performed sensitivity analysis to explore the potential sources of heterogeneity. And exclusion of any single study did not change the summarized results, which added robustness to our findings.

In this study, we found that the antibody-drug conjugate T-DM1 significantly improved PFS and OS among patients with HER2-positive MBC. Moreover, the subgroup analysis showed that T-DM1 provided a significant survival benefit for patient with HER2-positive MBC no matter it was used as first-line or non-first-line treatment. The TDM4450g study was a phase 2 trial [20], which directly compared the T-DM1 with an active HER2-target regimen for the first line treatment of HER2-positive MBC [20]. In that study, patients were randomly assigned to T-DM1 or trastuzumab plus docetaxel (HT) groups. The median PFS in the two groups was 14.2 months and 9.2 months, respectively (HR = 0.59, 95% CI: 0.36, 0.97) [20]. This indicated that T-DM1 was beneficial effect for patients with HER2-positive MBC when it was as first-line treatment [20]. Whereas in another three phase 3 trials [19, 21, 22], T-DM2 was administrated in patients who had previously treated with trastuzumab and lapatinib/taxnane. The median PFS among these studies ranged from 6.2 months to 9.6 months [19, 21, 22]. Thus, it was postulated that T-DM1 may provide better survival outcomes in patients who had never received standard treatment before than those who previously received anti-HER2 positive treatment. This hypothesis was verified in a phase 2 trial [17], in which T-DM1 was used as first-line and second-line treatment. Patients in the two groups had a median PFS of 7.7 months and 5.5 months, respectively, which indicated that T-DM1 would provide better survival effects for MBC patients when it was used as the first-line treatment [17].

The insensitivity to HER2-targeted therapies should be considered when deciding which agents to administer in the sequential treatments for HER2-positive MBC. Although HER2-targeted agents can inhibit the HER2 expression in tumor cells, the HER2-independent escape mechanisms, such as the constitutive activation of PI3K/AKT pathway, might lead to a less sensitive tumor phenotype [26, 27]. However, the favorable outcomes of T-DM1 in this meta-analysis supported the validity of HER2 as a therapeutic target in tumors that have progressed after several HER2-targeted therapies. Also, the beneficial effects of T-DM1 have been found in two phase 2 trials, in which MBC patients who received extensive pretreatment had improved PFS [15, 16]. In these two trials, T-DM1 was found to be more effective than treatment regimens that contained trastuzumab. This could be probably explained by the fact that the activity of T-DM1 was associated with high potency of its cytotoxic DM1 [13, 28], and it may be preserved in the presence of PI3K mutations [29].

With regard to the safety profile of T-DM1, this meta-analysis showed that patients receiving T-DM1 experienced more fatigue, elevated ALT, elevated AST, and thrombocytopenia than those receiving other anti-HER2 therapies; other anti-HER2 therapies were associated with more diarrhea, vomiting, neutropenia, leukopenia, and febrile neutropenia. Thrombocytopenia, was a rare but serious adverse event that occurred in the patients administrated with T-DM1. In the EMILIA trial [19], ten patients discontinued the treatment of T-DM1 because of thrombocytopenia, and one patient had a grade 4 bleeding event of gastrointestinal hemorrhage. Moreover, in the TH3RESA trial [22], a grade 5 hemorrhage event was observed in the T-DM1 group. Therefore, patients with thrombocytopenia should be monitored closely during the T-DM1 treatment.

There are some potential limitations in this meta-analysis. Firstly, our meta-analysis is based on five RCTs and some of them have a relatively modest sample size, which may lead to an overestimation of the treatment effect when compared with larger trials. Although all the included studies were well performed with a randomized controlled design and were high quality trial, our conclusion should be interpreted with caution. Secondly, the targeted population varied greatly across studies (e.g., ECOG PS, hormone-receptor status, treatment regimens, and line of therapy). These factors may cause the heterogeneity and have a potential impact on our final results. Lastly, it should be noted that all of these trials were partly funded by the pharmaceutical industry, and their results might have been affected by the inherent conflict of interest and possible bias.

In summary, our meta-analysis indicated that T-DM1 significantly improved PFS and OS in patients with HER-2positive MBC, and it also induced a higher incidence of adverse events. Thus, T-DM1 could be used as an alternate treatment option in patients with HER2-positive MBC, especially in those who had never received standard treatment before. Considering the potential limitations in this study, further larger-scale, well-design RCTs are needed to identify these findings.

## MATERIALS AND METHODS

### Search strategy

This meta-analysis was conducted in accordance with the Preferred Reporting Items for Systematic Reviews and meta-analysis (PRISMA) criteria [30]. Pubmed, Web of Science, and Embase databases from inception to May 18, 2017 were searched to identify relevant studies. The search was limited to human subjects and no language restriction was imposed. Search terms included: (“breast neoplasms”[MeSH Terms] OR (“breast”[All Fields] AND “neoplasms”[All Fields]) OR “breast neoplasms”[All Fields] OR (“breast”[All Fields] AND “cancer”[All Fields]) OR “breast cancer” [All Fields]) AND (“ado-trastuzumabemtansine” [Supplementary Concept] OR “ado-trastuzumabemtansine” [All Fields] OR “trastuzumabemtansine” [All Fields])). Details of search strategy are shown in Appendix 1. In addition, we also searched the reference lists of the included studies to identify other potentially eligible studies that we may left out with our primary search.

### Study selection

The following inclusive selection criteria were applied: (1) study design: randomized controlled trials (RCTs); (2) study population: female patients over the age of 18, who had histologically confirmed breast cancer with HER2-positive metastatic tumor; (3) comparison intervention: T-DM1 versus other anti-HER2 therapies; (4) outcome measure: the progression-free survival (PFS), overall survival (OS), overall response rate (ORR) and the adverse events. Studies published as the article types of reviews, editorials, letters, case report, and comments were excluded. In case that the same clinical trial appeared in several publications, we only included the most informative article or the longest follow-up study to avoid duplication of information. The inter-reviewer agreements were calculated using the Cohen K statistic [31].

### Data extraction and quality assessment

We used a standardized data-extraction sheet, which consisted of the following information: first author, publication year, number of patients in each arm, age of patients, population characteristics, the hazard ratios (HRs) with the corresponding 95% confidence intervals (CIs) on PFS and OS, and the risk rations (RRs) with the corresponding 95% CIs on incidence of adverse events. Data extraction was independently performed by two investigators, and discrepancies were resolved by discussion and consensus.

We used the method recommended by the Cochrane Collaboration [32] to assess the risk of bias in RCTs, including blinding, method of randomization, allocation concealment, follow-up, and intention-to-treat analysis. The quality of evidence for outcomes was evaluated according to the Grading of Recommendations Assessment, Development, and Evaluation (GRADE) [33]. This methodology consists of five items describing risk of bias, inconsistency, indirectness, imprecision, and publication bias [33]. The quality of each outcome is classified as very low, low, moderate, or high [33].

### Statistical analysis

We estimated the HR with 95% CI for time-to-event outcomes, and RR with 95% CI for dichotomous outcomes. Heterogeneity across the studies was tested using the Cochran Q statistic and quantified with the I^2^ statistic, in which I^2^ > 50% indicated significant heterogeneity [34]. whenever heterogeneity was present, a random-effects model [35] was used to pool the estimates, otherwise a fixed-effects model [36] was used. We also investigated the influence of a single study on the overall pooled estimate by deleting one study in each turn. Publication bias was assessed by the Begg's [24] and Egger's test [25]. A *P* value less than 0.05 was judged as statistically significant, except where otherwise specified. All statistical analyses were performed using STATA, version 12.0 (Stata Corporation, College Station, TX, USA).

## References

[1] Slamon DJ, Godolphin W, Jones LA, Holt JA, Wong SG, Keith DE, Levin WJ, Stuart SG, Udove J, Ullrich A, Press MF (1989). Studies of the HER-2/neu proto-oncogene in human breast and ovarian cancer. Science.

[2] Owens MA, Horten BC, Da Silva MM (2004). HER2 amplification ratios by fluorescence in situ hybridization and correlation with immunohistochemistry in a cohort of 6556 breast cancer tissues. Clin Breast Cancer.

[3] Slamon DJ, Leyland-Jones B, Shak S, Fuchs H, Paton V, Bajamonde A, Fleming T, Eiermann W, Wolter J, Pegram M, Baselga J, Norton L (2001). Use of chemotherapy plus a monoclonal antibody against HER2 for metastatic breast cancer that overexpresses HER2. N Engl J Med.

[4] Marty M, Cognetti F, Maraninchi D, Snyder R, Mauriac L, Tubiana-Hulin M, Chan S, Grimes D, Anton A, Lluch A, Kennedy J, O'Byrne K, Conte P (2005). Randomized phase II trial of the efficacy and safety of trastuzumab combined with docetaxel in patients with human epidermal growth factor receptor 2-positive metastatic breast cancer administered as first-line treatment: the M77001 study group. J Clin Oncol.

[5] Geyer CE, Forster J, Lindquist D, Chan S, Romieu CG, Pienkowski T, Jagiello-Gruszfeld A, Crown J, Chan A, Kaufman B, Skarlos D, Campone M, Davidson N (2006). Lapatinib plus capecitabine for HER2-positive advanced breast cancer. N Engl J Med.

[6] von Minckwitz G, du Bois A, Schmidt M, Maass N, Cufer T, de Jongh FE, Maartense E, Zielinski C, Kaufmann M, Bauer W, Baumann KH, Clemens MR, Duerr R (2009). Trastuzumab beyond progression in human epidermal growth factor receptor 2-positive advanced breast cancer: a german breast group 26/breast international group 03–05 study. J Clin Oncol.

[7] Blackwell KL, Burstein HJ, Storniolo AM, Rugo HS, Sledge G, Aktan G, Ellis C, Florance A, Vukelja S, Bischoff J, Baselga J, O'Shaughnessy J (2012). Overall survival benefit with lapatinib in combination with trastuzumab for patients with human epidermal growth factor receptor 2-positive metastatic breast cancer: final results from the EGF104900 Study. J Clin Oncol.

[8] Mohd Sharial MS, Crown J, Hennessy BT (2012). Overcoming resistance and restoring sensitivity to HER2-targeted therapies in breast cancer. Ann Oncol.

[9] Ahmed S, Sami A, Xiang J (2015). HER2-directed therapy: current treatment options for HER2-positive breast cancer. Breast Cancer.

[10] Remillard S, Rebhun LI, Howie GA, Kupchan SM (1975). Antimitotic activity of the potent tumor inhibitor maytansine. Science.

[11] Cassady JM, Chan KK, Floss HG, Leistner E (2004). Recent developments in the maytansinoid antitumor agents. Chem Pharm Bull (Tokyo).

[12] Widdison WC, Wilhelm SD, Cavanagh EE, Whiteman KR, Leece BA, Kovtun Y, Goldmacher VS, Xie H, Steeves RM, Lutz RJ, Zhao R, Wang L, Blattler WA (2006). Semisynthetic maytansine analogues for the targeted treatment of cancer. J Med Chem.

[13] Lewis Phillips GD, Li G, Dugger DL, Crocker LM, Parsons KL, Mai E, Blattler WA, Lambert JM, Chari RV, Lutz RJ, Wong WL, Jacobson FS, Koeppen H (2008). Targeting HER2-positive breast cancer with trastuzumab-DM1, an antibody-cytotoxic drug conjugate. Cancer Res.

[14] Junttila TT, Li G, Parsons K, Phillips GL, Sliwkowski MX (2011). Trastuzumab-DM1 (T-DM1) retains all the mechanisms of action of trastuzumab and efficiently inhibits growth of lapatinib insensitive breast cancer. Breast Cancer Res Treat.

[15] Burris HA, Rugo HS, Vukelja SJ, Vogel CL, Borson RA, Limentani S, Tan-Chiu E, Krop IE, Michaelson RA, Girish S, Amler L, Zheng M, Chu YW (2011). Phase II study of the antibody drug conjugate trastuzumab-DM1 for the treatment of human epidermal growth factor receptor 2 (HER2)-positive breast cancer after prior HER2-directed therapy. J Clin Oncol.

[16] Krop IE, LoRusso P, Miller KD, Modi S, Yardley D, Rodriguez G, Guardino E, Lu M, Zheng M, Girish S, Amler L, Winer EP, Rugo HS (2012). A phase II study of trastuzumab emtansine in patients with human epidermal growth factor receptor 2-positive metastatic breast cancer who were previously treated with trastuzumab, lapatinib, an anthracycline, a taxane, and capecitabine. J Clin Oncol.

[17] Miller KD, Dieras V, Harbeck N, Andre F, Mahtani RL, Gianni L, Albain KS, Crivellari D, Fang L, Michelson G, de Haas SL, Burris HA (2014). Phase IIa trial of trastuzumab emtansine with pertuzumab for patients with human epidermal growth factor receptor 2-positive, locally advanced, or metastatic breast cancer. J Clin Oncol.

[18] Perez EA, Hurvitz SA, Amler LC, Mundt KE, Ng V, Guardino E, Gianni L (2014). Relationship between HER2 expression and efficacy with first-line trastuzumab emtansine compared with trastuzumab plus docetaxel in TDM4450g: a randomized phase II study of patients with previously untreated HER2-positive metastatic breast cancer. Breast Cancer Res.

[19] Verma S, Miles D, Gianni L, Krop IE, Welslau M, Baselga J, Pegram M, Oh DY, Dieras V, Guardino E, Fang L, Lu MW, Olsen S (2012). Trastuzumab emtansine for HER2-positive advanced breast cancer. N Engl J Med.

[20] Hurvitz SA, Dirix L, Kocsis J, Bianchi GV, Lu J, Vinholes J, Guardino E, Song C, Tong B, Ng V, Chu YW, Perez EA (2013). Phase II randomized study of trastuzumab emtansine versus trastuzumab plus docetaxel in patients with human epidermal growth factor receptor 2-positive metastatic breast cancer. J Clin Oncol.

[21] Welslau M, Dieras V, Sohn JH, Hurvitz SA, Lalla D, Fang L, Althaus B, Guardino E, Miles D (2014). Patient-reported outcomes from EMILIA, a randomized phase 3 study of trastuzumab emtansine (T-DM1) versus capecitabine and lapatinib in human epidermal growth factor receptor 2-positive locally advanced or metastatic breast cancer. Cancer.

[22] Krop IE, Kim SB, Gonzalez-Martin A, LoRusso PM, Ferrero JM, Smitt M, Yu R, Leung AC, Wildiers H (2014). Trastuzumab emtansine versus treatment of physician's choice for pretreated HER2-positive advanced breast cancer (TH3RESA): a randomised, open-label, phase 3 trial. Lancet Oncol.

[23] Perez EA, Barrios C, Eiermann W, Toi M, Im YH, Conte P, Martin M, Pienkowski T, Pivot X, Burris H, Petersen JA, Stanzel S, Strasak A (2017). Trastuzumab Emtansine With or Without Pertuzumab Versus Trastuzumab Plus Taxane for Human Epidermal Growth Factor Receptor 2-Positive, Advanced Breast Cancer: Primary Results From the Phase III MARIANNE Study. J Clin Oncol.

[24] Valle PM, Mosegui GB, Vianna CM, Araujo RL, Felicissimo T, Lima IJ (2015). Trastuzumab Emtansine for Her2 Positive Breast Cancer Patients: an Updated Systematic Review. Value Health.

[25] Shen K, Ma X, Zhu C, Wu X, Jia H (2016). Safety and Efficacy of Trastuzumab Emtansine in Advanced Human Epidermal Growth Factor Receptor 2-Positive Breast Cancer: a Meta-analysis. Sci Rep.

[26] Berns K, Horlings HM, Hennessy BT, Madiredjo M, Hijmans EM, Beelen K, Linn SC, Gonzalez-Angulo AM, Stemke-Hale K, Hauptmann M, Beijersbergen RL, Mills GB, van de Vijver MJ (2007). A functional genetic approach identifies the PI3K pathway as a major determinant of trastuzumab resistance in breast cancer. Cancer Cell.

[27] Wang L, Zhang Q, Zhang J, Sun S, Guo H, Jia Z, Wang B, Shao Z, Wang Z, Hu X (2011). PI3K pathway activation results in low efficacy of both trastuzumab and lapatinib. BMC Cancer.

[28] Lopus M, Oroudjev E, Wilson L, Wilhelm S, Widdison W, Chari R, Jordan MA (2010). Maytansine and cellular metabolites of antibody-maytansinoid conjugates strongly suppress microtubule dynamics by binding to microtubules. Mol Cancer Ther.

[29] Baselga J, Verma S, Ro J Relationship between tumor biomarkers (BM) and efficacy in EMILIA, a phase III study of trastuzumab emtansine (T-DM1) in HER2-positive metastatic breast cancer (MBC).

[30] Moher D, Liberati A, Tetzlaff J, Altman DG (2009). Preferred reporting items for systematic reviews and meta-analyses: the PRISMA statement. Ann Intern Med.

[31] Sim J, Wright CC (2005). The kappa statistic in reliability studies: use, interpretation, and sample size requirements. Phys Ther.

[32] Higgins JP, Altman DG, Gotzsche PC, Juni P, Moher D, Oxman AD, Savovic J, Schulz KF, Weeks L, Sterne JA (2011). The Cochrane Collaboration's tool for assessing risk of bias in randomised trials. Bmj.

[33] Guyatt GH, Oxman AD, Vist GE, Kunz R, Falck-Ytter Y, Alonso-Coello P, Schunemann HJ (2008). GRADE: an emerging consensus on rating quality of evidence and strength of recommendations. Bmj.

[34] Higgins JP, Thompson SG (2002). Quantifying heterogeneity in a meta-analysis. Stat Med.

[35] Cochran WG (1954). The combination of estimates from different experiments. Biometrics.

[36] DerSimonian R, Laird N (1986). Meta-analysis in clinical trials. Control Clin Trials.

